# Does skeletal muscle have an ‘epi’‐memory? The role of epigenetics in nutritional programming, metabolic disease, aging and exercise

**DOI:** 10.1111/acel.12486

**Published:** 2016-04-22

**Authors:** Adam P. Sharples, Claire E. Stewart, Robert A. Seaborne

**Affiliations:** ^1^ Stem Cells, Ageing and Molecular Physiology (SCAMP) Research UnitExercise Metabolism and Adaptation Research Group (EMARG)Research Institute for Sport and Exercise Sciences (RISES)Liverpool John Moores UniversityLiverpoolUK

**Keywords:** ageing, aging, aging muscle, beta‐catenin, cellular programming, developmental programming, DNA methylation, epigenetics, exercise, fibre number, fibre type, foetal programming, forkhead box, healthspan, histone acetylation, histone deacetylation, histone modification, insulin‐like growth factor, lifespan, metabolic programming, muscle memory, muscle precursor cell, muscle stem cell, myf5, myoblast, myocyte, myoD, myogenesis, myogenin, myogenic regulatory factor, MRF4, NFKB, nutritional programming, obesity, sarcopenia, tumour necrosis factor alpha, type II diabetes, myostatin

## Abstract

Skeletal muscle mass, quality and adaptability are fundamental in promoting muscle performance, maintaining metabolic function and supporting longevity and healthspan. Skeletal muscle is programmable and can ‘remember’ early‐life metabolic stimuli affecting its function in adult life. In this review, the authors pose the question as to whether skeletal muscle has an ‘epi’‐memory? Following an initial encounter with an environmental stimulus, we discuss the underlying molecular and epigenetic mechanisms enabling skeletal muscle to adapt, should it re‐encounter the stimulus in later life. We also define skeletal muscle memory and outline the scientific literature contributing to this field. Furthermore, we review the evidence for early‐life nutrient stress and low birth weight in animals and human cohort studies, respectively, and discuss the underlying molecular mechanisms culminating in skeletal muscle dysfunction, metabolic disease and loss of skeletal muscle mass across the lifespan. We also summarize and discuss studies that isolate muscle stem cells from different environmental niches *in vivo* (physically active, diabetic, cachectic, aged) and how they reportedly remember this environment once isolated *in vitro*. Finally, we will outline the molecular and epigenetic mechanisms underlying skeletal muscle memory and review the epigenetic regulation of exercise‐induced skeletal muscle adaptation, highlighting exercise interventions as suitable models to investigate skeletal muscle memory in humans. We believe that understanding the ‘epi’‐memory of skeletal muscle will enable the next generation of targeted therapies to promote muscle growth and reduce muscle loss to enable healthy aging.

## Scope and aims of the review

For the purposes of this review, the author defines skeletal muscle memory as:

The capacity of skeletal muscle to respond differently to environmental stimuli in an adaptive or maladaptive manner if the stimuli have been previously encountered.

We therefore suggest that skeletal muscle memory refers to both cellular and tissue retention of prior environmental stimuli or stress such as those from acute or chronic exercise, muscle damage/injury, disease or changes in nutrients which culminate in altered behaviour, if the stimulus is re‐encountered. Ultimately, this is important for skeletal muscle because if the environment encountered is an anabolic or positive one, muscle may respond to these later‐life stimuli with additive muscle growth or healthy maintenance across the lifespan; however, if the environment is catabolic or negative, it may become more susceptible to muscle mass loss in later life. This therefore has important consequences, as sufficient quantity and quality of skeletal muscle are required not only to sustain performance in elite sport but also to enhance lifespan and promote healthspan into older age [recently reviewed in Sharples *et al*. (2015b)].

Historically, the term ‘programming’ has been used to define similar processes of ‘memory’ in cells or tissues. Programming however, can evoke a slightly different perception in the minds of scientists even within similar biological fields. For example, programming in embryonic stem cell biology refers to the programme that controls pluripotency in embryonic stem (ES) cells, and reprogramming is a human intervention whereby adult cells are returned to pluripotent embryonic‐like states, where any previous environmental encounters experienced during development or with age are essentially re‐set. This is therefore somewhat distinct from tissue or cells retaining information after a stimulus or stress in readiness for future stimuli that when re‐encountered may bring about further adaptation, or maladaptation. Metabolic or nutrient programming is another example of a phenomenon linked, yet somewhat distinct to what we define as skeletal muscle memory above. This usually refers to a change in nutritional stimuli such as global calorie or protein restriction or a high‐fat diet that changes physiology and metabolism of the organism during development that is usually then fixed into older postnatal age. Therefore, these adaptations continue even without confrontation with the same stimulus/stress that initiated them. The majority of work in the metabolic programming field is undertaken using *in utero* studies, therefore also termed foetal or developmental programming, where the offspring encounter different environmental stimuli during pregnancy and the phenotypes postbirth (sometimes into adulthood or old age) are dependent on these earlier life occurrences. This field provided some of the earliest evidence to suggest that skeletal muscle was programmable and evolved into the research field encompassing foetal origins of health and disease. Given that this particular field is beginning to examine the molecular and epigenetic mechanisms of these phenomena and provide insights into the concept of muscle memory defined above, this literature is reviewed below.

In addition to the skeletal muscle being programmable, there is also now emerging evidence that suggests even after short‐term environmental stimuli, skeletal muscle can retain molecular information in order to be primed for future plasticity following encounters with the same stimulus. Just as we do when we suddenly recall a past childhood memory after being in contact with the same or similar stimuli in adulthood, we propose that skeletal muscle could behave within a similar conceptual framework *via* distinct molecular mechanisms. We suggest that epigenetics, in particular, could mechanistically underpin muscle memory as defined above; therefore, we present the term muscle ‘epi’*‐memory* in this review to capture this notion.

**Figure 1:**
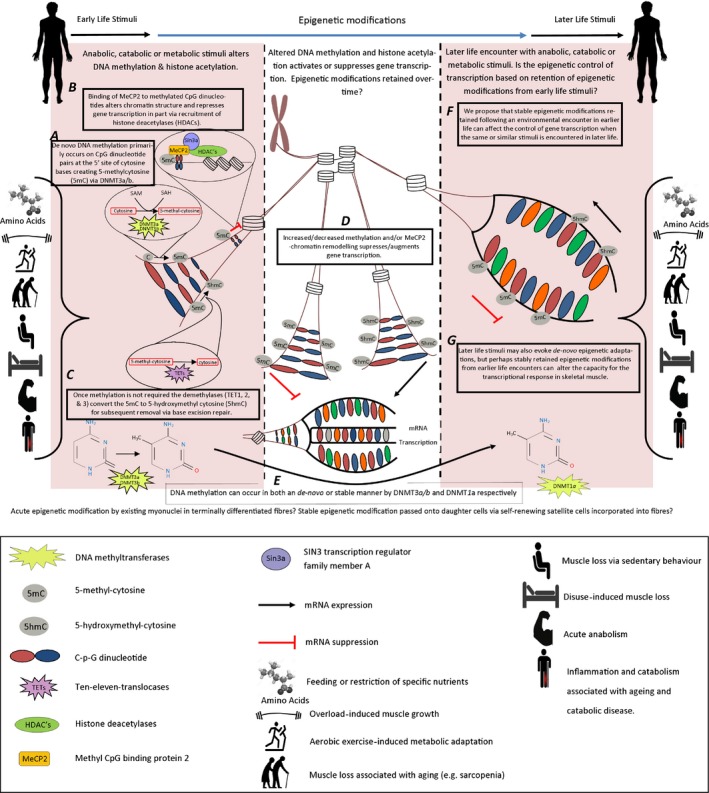
A proposed schematic representation of skeletal muscle ‘epi’‐memory.

Epigenetics is defined as the study of changes in organisms caused by the modification of gene expression rather than the alteration of the genetic code itself (Oxford Dictionary, 2015). Because environmental stimuli, stresses or encounters cause modifications in gene expression, turning genes on or off, and muscle memory is defined as the capacity for skeletal muscle to respond or adapt differently to environmental stimuli if encountered previously, it suggests that epigenetics could mechanistically underpin skeletal muscle memory. Epigenetic modifications can be extremely transient, for example after a simple bout of acute metabolic stress (high‐intensity aerobic exercise) (Barres *et al*., 2012), or obstinately stable, termed genetic inheritance, even being passed to daughter generations of cells (Hansen *et al*., 2008; Ng & Gurdon, 2008; Petruk *et al*., 2012). Critically, it has been reported that epigenetic DNA marks maintained in germlines and even RNAs can be transferred to the next generation in mammals (Anway *et al*., 2005; Campos *et al*., 2014; Gapp *et al*., 2014; Liebers *et al*., 2014; Grandjean *et al*., 2015; Sharma *et al*., 2016), contributing to ‘heritable’ metabolic disease (Grandjean *et al*., 2015; Chen *et al*., 2016; Donkin *et al*., 2016). This evidence firmly points to epigenetics playing an important role in the retention of information into later life. Finally, our group has recently shown that skeletal muscle cells retain epigenetically modified DNA ‘tags’ (methyl groups) as a result of early‐life inflammatory stress and that they seemingly retain this information until later in their proliferative lifespan *in vitro* (Sharples *et al*., 2015a), a concept that is discussed later in this review (and depicted in Fig. 1). We therefore believe that skeletal muscle memory is underpinned by epigenetic modifications (‘epi’‐memory).

Therefore, in this review, we will; (i) outline the emergence of scientific evidence contributing to the notion of skeletal muscle memory including metabolic/foetal programming studies and the underlying molecular mechanisms; (ii) discuss the importance of memory/programming in metabolic disease and the underlying molecular mechanisms; (iii) review the data within epidemiological research where aging cohort studies support skeletal muscle programming in humans; (iv) summarize and discuss the evidence for muscle memory from animal tissue models and where muscle stem cells have been isolated under different environmental niches *in vivo,* for example physically active, diabetic, cachexic and aged, and how they ‘memorize’ this environment once isolated *in vitro*; (v) finally, we will outline the proposed molecular and epigenetic mechanisms underlying skeletal muscle memory and review the epigenetic regulation of exercise‐induced skeletal muscle adaptation, highlighting exercise interventions as suitable models to investigate skeletal muscle memory.

## Emergence of evidence for skeletal muscle memory

### Early‐life nutrient restriction and the programming of skeletal muscle fibre number, composition and size

The Dutch famine (1944–1945), affecting women in the first trimester of pregnancy, was associated with an increased prevalence of coronary heart disease, raised lipids and obesity in the offspring. Severe malnourishment during late gestation was also related to impaired glucose tolerance of the offspring into adult life (Ravelli *et al*., 1998, 1999; Roseboom *et al*., 2000a,b). The influence of this catastrophic event was only fully realized in the late 1990s as these children were tracked into adulthood; it had, however, been observed some 16 years previously that malnutrition during pregnancy in rats reduced general cell numbers in the offspring (McLeod *et al*., 1972). These studies resulted in a programme of research in the field of foetal/developmental programming (later also defined as metabolic/nutritional programming), with basic and clinical scientists examining how these early‐life encounters impacted health and function or dysfunction into adulthood and old age. Follow‐up animal studies have reported nutritional programming in the liver (Gluckman *et al*., 2007), kidney, lung (Brameld *et al*., 2000; Yakubu *et al*., 2007a,b), adipose tissue (Budge *et al*., 2003), the brain (Guzman‐Quevedo *et al*., 2013) and even skeletal muscle. More recently, a smaller subgroup of individuals (150 men and women) from the Dutch famine birth cohort (aged 68) were reported to display increased mortality and were more frequently admitted to hospital for age‐related disease. In the same study, at the organ/system level, the authors demonstrated larger increases in levels of aging biomarkers in the brain, bone, skeletal muscle and the eye. At the cellular level, increased inflammation, oxidative stress and decreased telomere length of DNA, a hallmark of cellular senescence, were evident (de Rooij & Roseboom, 2013). Overall, this evidence has led to the emergence of the notion that, even decades later, organs, tissues and even cells can be programmed as a result of early‐life foetal environmental encounters.

Skeletal muscle tissue is the largest metabolic organ. Therefore, skeletal muscle was an early target of assessment in metabolic programming studies. It is important to note that the gestational nutritional manipulations implemented in experiments detailed in this review occur at different times and for differing durations, and this may impact on data interpretation when attempting to compare across experiments or to draw conclusions. It is therefore important to understand the time course of developing skeletal muscle in the different mammalian models used. It is also important to appreciate that the majority of studies in the mammalian developmental programming field are undertaken in sheep due to their similar pregnancies to humans. The first fibres formed in skeletal muscle development and growth are called primary muscle fibres and in mammals begin to develop at 32 days during gestation and continue to form up to approximately 38 days. Primary myofibres have myofibrils that are peripherally located and surround an axial core of nuclei and cytoplasm (Beermann *et al*., 1978). Secondary fibres then use the primary fibres as a scaffold and develop after 38–62 days (Wilson *et al*., 1992). They are derived from PAX7‐positive precursor cells that become activated myoblasts that terminally fuse/differentiate into myotubes and subsequently mature into muscle fibres parallel to the primary fibres, and go on to make up the majority of skeletal muscle. This process is driven by a dramatic downregulation of PAX7 and sequential activation of muscle‐specific myogenic regulatory factors (MRFs), myf5 (proliferation), myoD (early fusion), myogenin (fusion) and mrf4 (late fusion/myotube maturation) (as reviewed in Sabourin & Rudnicki, 2000; Maltin *et al*., 2001). This is followed by an advancement of tertiary myofibre development from 62 days (Wilson *et al*., 1992). In rodents, the primary fibres develop at 14–16 days, secondary fibres around 17–19 days (Wilson *et al*., 1988) and tertiary formation thereafter. Generally, the primary fibres become slow (type I/oxidative), whereas secondary fibres become fast fibres (type IIA: fast, mixed oxidative and glycolytic and type IIB: fast, glycolytic) (Draeger *et al*., 1987).

To the authors’ knowledge, it was first observed that prenatal nutrition negatively affected skeletal muscle fibre growth and composition in the offspring, where a 30% reduction in total calories consumed reduced the number of fast fibres in both* *the soleus and lumbrical* *muscles of newborn rats (Wilson *et al*., 1988). Importantly, re‐feeding of the mothers during lactation was unable to restore fibre number in the soleus of restricted offspring vs. controls, yet was able to restore fibre number in the lumbrical muscle (Wilson *et al*., 1988). This suggested that fibre number within the rat soleus was fixed into older age, but this effect was fibre type dependent. It was later confirmed in other species (guinea pig) that 40–60% total nutrient restriction throughout gestation reduced birth weight of the offspring and reduced myofibre number in the biceps brachii by 20–26% (Ward & Stickland, 1991; Dwyer & Stickland, 1994; Dwyer *et al*., 1995). Together with a reduction in fibre number, it has been demonstrated that global nutrient restriction during pregnancy can also impact on the composition (fibre type) and size of the fibre. In ewes, 50% globally reduced maternal nutrition between 30–70 days of gestation resulted in fewer fast and increased slow fibre numbers in the vastus lateralis, 14 days postpartum, vs. relevant controls, an observation notwithstanding in the group restricted at 30–70 days or between 55–95 days (Fahey *et al*., 2005). Furthermore, 50% nutrient restriction in the conception period (minus 6–18 days of gestation) reduced myofibre number of the mid‐gestation foetus but had no effect on foetal weight (Quigley *et al*., 2005). Therefore, it appears that the impact of nutrient restrictions on fibre type, composition and size is gestation dependent, with a general view that earlier restriction impacts fibre number, mid‐gestation impacts fibre composition (predominantly fast fibres) and later restriction impacts muscle size and weight. However, to complicate this observation, it has been subsequently shown that a similar, yet longer phase of global restriction (50%) in ewes between 28 and 70 days of gestation actually increased fast myosin type IIb isoforms, albeit on a background of reduced total myofibre number, in the longissimus dorsi of 8‐month‐old offspring (Zhu *et al*., 2006). It is therefore worth postulating that adaptations are dependent on timing, duration, degree of restriction and the muscles impacted, for example, the triceps brachii (both slow‐ and fast‐twitch myofibres) suffer reduced fibre and capillary density in the offspring of restricted ewes in response to restricted maternal diet vs. no change in the soleus (majority slow‐twitch myofibres) under the same conditions (Costello *et al*., 2008). Coupled with altered fibre number, composition and muscle size, reduced maternal nutrition during 28–78 days of gestation in ewes results in increases in fat mass (both subcutaneous and visceral) and reduced lean mass in the offspring (Ford *et al*., 2007). The translation to human studies however, is somewhat compounded, in that women who are lean and on a restricted diet during pregnancy are generally speaking also equivalently lean and restricted pre‐pregnancy. Therefore, to simulate this situation, researchers investigated the impact of low (excessively lean) maternal body scores (LBS) before and throughout gestation in ewes vs. high body score (HBS) ewes (obese) (Costello *et al*., 2013). Here, bioptic samples highlighted impairments in the skeletal muscle of the offspring produced by LBS mothers, including reductions in total myofibre density, fast fibre size and capillary‐to‐myofibre ratio (Costello *et al*., 2013), observations similar to those in nutrient‐restricted studies. While questions remain and specifics need to be determined, overall, it is clear that as a consequence of nutrient restriction *in utero,* skeletal muscle demonstrates the alterations in fibre number, composition and size, which prevail into later life, despite normal feeding patterns.

### Maternal protein restriction and skeletal muscle programming

The majority of the studies above manipulated nutritional intake *via* a 40–60% global reduction in food consumed. Some studies have also altered specific macronutrients to measure the impact of individual nutritional components on metabolic programming. With respect to skeletal muscle, as protein feeding is important in protein synthesis and muscle maintenance across the lifespan, a particular interest has focused on protein‐restricted rodent maternal diets, where rats that had their protein intake restricted (9% vs. 18% in controls as a proportion of total matched calories from protein) during mid‐pregnancy (8–14 days in rats), demonstrated significantly reduced muscle fibre number and density in their 4‐week‐old offspring (Mallinson *et al*., 2007). As with the restriction of total energy, these observations were fibre type and muscle group dependent where the number and density of fast‐twitch fibres in the soleus were found to be reduced in the offspring after early (0–7 days), mid (8–14 days)‐ and late (15–22 days) restriction, whereas in the gastrocnemius, only a reduction in the density of slow‐twitch fibres occurred at mid (8–14 days)‐restriction (Mallinson *et al*., 2007). A more recent study suggested that a more severe maternal protein restriction at mid (7–14 days)‐gestation (6% of total calories from protein vs. 17% in control group) reduced type I fibre diameter by 20% together with a reduction in type IIa fibres by 5%, while type IIb fibres actually increased by 5% vs. the aged matched control group in aged rat offspring (365 days old) (Confortim *et al*., 2015). Finally, in the same study it was shown that protein restriction during gestation results in a reduction in size of neuromuscular junctions; as age‐related decline in muscle is associated with denervation (Confortim *et al*., 2015), these fascinating findings suggest an important role for early‐life encounters on muscle size and function into old age, which is discussed later in this review. Therefore once again, although there are some discrepancies in the specific fibre‐type changes following protein restriction, on a background of differences in muscle groups studied, timing of protein restriction and sampling of the offspring, it is clear that the general consensus is that muscle size is reduced following maternal protein restriction. The important role of protein per se has been illustrated in guinea pig models, where all dietary components were reduced to 60% of the *ad libitum* level, but pregnant mothers were supplemented with protein, to the same level as controls during gestation. It was reported that muscle fibre number in the offspring returned back to the level of the *ad libitum*‐fed controls (Dwyer & Stickland, 1994). This however, is also observed when carbohydrates are supplemented back, but not fat (Dwyer & Stickland, 1994), suggesting that at least in this study, the different macronutrients have an equal role in the programming of fibre number. Further, supplementing with higher protein (55% vs. 20% in control) during rat gestation and weaning reduces birth weight by 7% by a reduction in adipose tissue (31%) (Desclee de Maredsous *et al*., 2016). Overall these studies suggest that programming due to protein restriction may be somewhat reversible by protein feeding during gestation or lactation.

### Proposed molecular mechanisms of reduced muscle size under maternal nutrient restriction

Although a vast number of studies contribute to what we know about morphological and compositional adaptations of skeletal muscle into adulthood and following early‐life nutrient stress, there are few studies to investigate the potential molecular mechanisms of metabolic programming. However, foetuses of globally (50%) nutrient‐restricted mothers have a reduced activity of protein synthetic pathways, including a reduced activation of mammalian target of rapamycin (mTOR) at Ser2448 and ribosomal protein S6 (p70S6K) at Ser235/336 with corresponding reductions in fibre size and the number of secondary fibres (Zhu *et al*., 2004). The alterations in protein activity in this study reportedly occurred without the change in the total levels of the signalling proteins, energy sensing activity (e.g. 5′ adenosine monophosphate‐activated protein kinase/AMPK) or protein degradative signalling (Zhu *et al*., 2004). However, reductions in upstream protein kinase B (PKB or Akt) total protein levels were observed in offspring from LBS mothers (Costello *et al*., 2013). Further, upstream of this intracellular signalling, it has also been observed that restricted global maternal nutrition (60%) altered gene expression of the ligand, insulin‐like growth factor II (IGF‐II), in the skeletal muscle of sheep fetuses sampled at 80 days gestation (Brameld *et al*., 2000). In this study, IGF‐I was found to be not altered locally in the skeletal muscle, but was reduced in production by the liver. It may be hypothesized that greater IGF‐II may have actually led to earlier and enhanced differentiation in muscle during gestation, causing a disruption of myofibre number. This, however, is only speculative as overexpression of IGF‐II by our group has been shown to have this role in adult muscle cells (Stewart *et al*., 1996) and not as yet, developing muscle tissue. Alternatively, this could be due to a compensatory mechanism to attempt to promote mesoderm growth (Morali *et al*., 2000) in the face of nutrient restriction, especially when it has been previously observed in work by our group that IGF‐IIR knockout increased circulating IGF‐II and the mice were 25–30% larger vs. controls (Lau *et al*., 1994). A recent study suggested that the reduction in muscle size seen in the offspring of rat mothers subjected to protein restriction (8% total calories vs. 20% total calories) was also due to the reductions in amino acid response (AAR) pathway‐related genes and an elevation of autophagy‐related genes. Interestingly, in this study, the authors alluded to the concept of muscle memory stating that the muscle at 38 days postpartum had ‘memorized’ the early‐life low‐protein environment (Wang *et al*., 2015). Furthermore, a low‐protein maternal diet in pigs increased the gene expression of the ligand myostatin and its receptor (activin type II receptor) in 35‐day‐old offspring (Liu *et al*., 2015). Myostatin is a potent negative regulator of muscle mass (McPherron & Lee, 1997); therefore, these studies add to the growing data set, suggesting that alterations in protein synthetic/degradative pathways *in utero* impact on muscle composition in later life (Liu *et al*., 2015).

It has also been proposed that glucose‐restricted sheep (40%) have decreased protein accretion, driven not by reductions in protein synthesis, a finding that is in opposition to Zhu *et al*., (2004) and Costello *et al*. (2013), but by an increase in protein degradation *via* the skeletal muscle ubiquitin‐proteasome pathway (Brown *et al*., 2014). In this study, increased mRNA production of atrogin 1 (MAFbx1) and muscle ring finger 1 (MuRF1) (Brown *et al*., 2014) were reported, both ubiquitin ligases important in the tagging of proteins for degradation. No alterations, however, were observed in autosome–lysosome pathways, for example mRNA for cathepsin L1, BCL2/adenovirus E1B 19‐kDa protein‐interacting protein *(BNIP3)* or beclin 1. It is also worth mentioning when trying to account for these potential discrepancies that the muscle of these offspring in studies by Costello *et al*. (2013) and Zhu *et al*., (2014) were sampled after gestation into adulthood, whereas the study by Brown *et al*. (2014) sampled 8‐week‐old foetuses. Furthermore, the studies by Costello *et al*., 2013 and Zhu *et al*., 2014 showed a reduction in protein synthetic signalling, via the manipulation of total nutritional content across all macronutrients, whereas glucose was the only nutrient restricted when ubiquitin‐proteasome pathway was impaired (Brown *et al*., 2014). Therefore, with the caveat that not all studies investigated protein degradation, it would perhaps be suitable to hypothesize that a reduction in other macronutrients (e.g. protein, as this usually makes up the other largest macronutrient to be restricted) may, as expected, drive the reductions in protein synthesis (e.g. *via* mTOR), whereas the reductions in glucose (carbohydrate) may drive increases in protein degradation, something that has previously been observed in starvation in the skeletal muscle cells *via* the activation of forkhead box **(**FOXO) transcription factors leading to the activation of atrogin 1 gene expression and subsequent protein degradation (Sandri *et al*., 2004). However, the role of FOXO in nutritional programming requires further investigation in skeletal muscle during nutrient restriction. To the authors’ knowledge, it has only been investigated in the offspring of maternally obese sheep who are overnourished. Here, sheep consumed 150% of the recommended global nutrient intake for 60 days prior to conception and 75 days into gestation at which point the foetal muscle was sampled (Tong *et al*., 2009). The sampled muscle demonstrated increased FOXO3a and nuclear factor kappa light chain enhancer of activated B cells (NF‐κB) signalling (via increased IkappaB kinase (IKKβ) and p65 activity). Due to FOXO's role in mediating NF‐κB‐driven inflammation (van der Horst & Burgering, 2007) in the skeletal muscle [reviewed in Li *et al*. (2008)] and obesity [reviewed in Tornatore *et al*. (2012)], as well as its role in quiescence mammalian cells (Bowerman, 2005) and muscle atrophy (Sandri *et al*., 2004), the authors hypothesized that FOXO could be involved in the reduction of myogenesis observed in the foetal muscle of obese mothers. Indeed, they found that the foetal muscle had reduced fibre size and reductions in MRFs controlling myogenesis *via* the reduced content of myoD and myogenin as well as the intermediate protein filament desmin and, in an earlier study by the same group, increased adipogenesis (Zhu *et al*., 2008). This drop in myogenesis was observed with reduced beta‐catenin, which has been shown to be involved in the activation of transcription of the above MRFs (Cossu & Borello, 1999). As FOXO diverts beta‐catenin away from the nucleus where it is unable to form an active transcription complex with members of the TCF family of transcription factors involved in the transcription of the MRFs (Almeida *et al*., 2007), these findings suggested that FOXO may be involved in mediating the reductions in myogenesis and fibre size in the foetal muscle. Although protein degradation was not directly analysed in this study, the observations point to a potentially important role for FOXO in the programming effect in skeletal muscle following early‐life nutrient stress. NF‐κB's signalling has also been shown to be involved in atrophy of skeletal muscle *via* protein degradation (reviewed in Jackman & Kandarian, 2004; Bakkar *et al*., 2008), but also plays a role in early muscle cell survival where NF‐κB inhibition in the presence of an apoptotic dose of tumour necrosis factor‐α (TNF‐α) exacerbates cell death (Stewart *et al*., 2004). Furthermore, as both NF‐κB and FOXO have been linked with cell survival instead of growth, that is, shifting cellular function towards oxidative stress resistance and DNA repair (Brunet *et al*., 2004; Greer & Brunet, 2005; Wang *et al*., 2007), and are associated with lifespan extension (Giannakou *et al*., 2004; Alic *et al*., 2014), these molecules could be fundamental in programming and perhaps memory, particularly impacting in later life where impaired DNA repair and oxidative stress are linked with the muscle loss and sarcopenia (reviewed in Jackson & McArdle, 2011). This is especially relevant when maternal obesity in ewes has been shown to increase fibrogenesis vs. skeletal muscle tissue accretion in the foetal skeletal muscle (Tong *et al*., 2009), a hall mark of sarcopenia, and one of the fundamental causes of reduced muscle force and quality (force per cross‐sectional area of muscle) (reviewed in Serrano & Munoz‐Canoves, 2010; Mann *et al*., 2011). It is also worth noting that as well as protein restriction, high‐fat gestational rodent feeding and postpartum rodent feeding have been shown to reduce protein synthetic signalling *via* p70S6 kinase 1 and 4E binding protein‐1 phosphorylation while also manipulating FOXO and NF‐&kgr;B action. Overall, these studies suggest that the mechanisms for reduced muscle size following maternal nutrient restriction involve reductions in protein synthetic signalling and increases in protein degradative signalling, with the latter perhaps more likely when carbohydrates alone are restricted. High‐fat diets are also detrimental to muscle size *via* reduced myogenic cues, reduced protein synthetic signalling, increased protein degradation and enhanced inflammation.

### Nutrient/metabolic programming and the impact on metabolism and metabolic disease

Metabolic programming is also clinically important, as it has been suggested that early‐life stress‐induced alterations in skeletal muscle fibre type and size may predispose individuals to metabolic abnormalities such as obesity (Tanner *et al*., 2002) and type II diabetes (Marin *et al*., 1994). Indeed, nutrient restriction during mid‐ and late gestation (but not early gestation) results in glucose intolerance in response to an intravenous glucose tolerance test (IGTT) (Gardner *et al*., 2005; Ford *et al*., 2007). It is worth noting that in this study this phenomena may not have not been due to muscle tissue but may have been a result of reduced adipose tissue disposal of glucose *via* reductions in insulin‐responsive glucose transporter 4 (GLUT4), an observation that was not found in the skeletal muscle. However, the glucose intolerance observed was not related to insulin deficiency as insulin area under the curve in response to an IGTT was actually increased in the offspring of global restricted maternal nourishment (Gardner *et al*., 2005). Furthermore, in the skeletal muscle specifically, a key enzyme controlling fat oxidation, carnitine palmitoyltransferase‐1, has been shown to be reduced by approximately 25% in the skeletal muscle of ewe offspring from nutrient‐restricted parents, where intramuscular triglyceride content (IMTG) was also found to be increased contributing to an increased fat deposition (Zhu *et al*., 2006). As increased IMTG content predisposes insulin resistance in the skeletal muscle (Phillips *et al*., 1996; Krssak *et al*., 1999), these findings are suggestive of an increased likelihood of diabetes. Furthermore, rats considered small for their gestational age (Deng *et al*., 2014) and humans with low birth weight (Ozanne *et al*., 2005) can also develop insulin resistance and glucose intolerance. Indeed, it was observed that in Danish low birth weight men, levels of GLUT4 protein abundance and insulin signalling [protein kinase C zeta (PKCz) and the p85a/p110b subunits of phosphoinositol‐3‐kinase)] were found to be reduced in their skeletal muscle (Ozanne *et al*., 2005), overall pointing to an altered glucose handling and a predisposition to type II diabetes in response to early foetal environmental encounters. To relate this altered phenotype of low birth weight in humans to foetal nutrient restriction, in the same study, rats were exposed to low‐protein diets during gestation and lactation, where the rats exhibited changes similar to the Danish men with low birth weights (Ozanne *et al*., 2005). Later studies have also confirmed the above findings, where gestational low protein (6% vs. 20% in control) in rats led to insulin resistance in adult offspring by involving an inadequate insulin‐induced phosphorylation of the Insulin receptor, insulin receptor substrate‐1 (IRS‐1) and Akt substrate of 160 kDa (AS160) as well as an impaired GLUT4 translocation and the corresponding increases in glucose and insulin levels when subjected to an IGTT (Blesson *et al*., 2014). Indeed, maternal low protein (8%) and postnatal high diets (45%) in the rats have been shown to increase the risk of type II diabetes by decreasing skeletal muscle oxidative respiration, *via* increased sirtuin 3 (Sirt3), and potentially by decreasing the activity of succinate dehydrogenase (SDH) (Claycombe *et al*., 2015). Protein restriction in maternal rat and subsequent acute fasting of the resulting offspring can also alter the metabolic properties of faster muscle fibres (EDL vs. soleus) (Aragao *et al*., 2014). It was observed in this study that fasting in the offspring on a background of maternal protein restriction reduced the gene expression of lipid metabolism regulators peroxisome proliferator‐activated receptors alpha (PPARα), PPARδ and peroxisome proliferator‐activated receptor gamma coactivator 1‐alpha (PGC‐1α – an important regulator of mitochondrial biogenesis and carbohydrate/lipid metabolism) to the same extent as nonfasted controls in their slow‐twitch (soleus) muscles, yet, in faster fibres (EDL) control animals were protected from fasting‐induced reductions in gene expression, whereas the protein‐restricted group demonstrated impairments, a phenomenon that the authors described as metabolic ‘inflexibility’ (Aragao *et al*., 2014). However, it was clear that in this study the fast‐twitch muscle was able to retain information from early‐life encounters with nutrient stress and was subsequently more susceptible to later‐life stress of the same kind, demonstrating skeletal muscle memory.

Overall, to substantiate that nutritional programming influences metabolic inadequacies; hypertension, insulin resistance and obesity have been observed in the adult offspring of obese C57BL/6J mice (Samuelsson *et al*., 2008; Shelley *et al*., 2009). Also, the skeletal muscles from foetuses from obese sheep have blunted insulin and AMPK signalling as well as an increased inflammatory cytokine tumour necrosis factor‐alpha (TNF‐α), in their plasma, suggesting the development of insulin resistance in these offspring (Zhu *et al*., 2008). Somewhat counterintuitively however, high‐fat diets during gestation in pigs have been seen to increase myofibre cross‐sectional area (Fainberg *et al*., 2014). However, pigs are encouraged to eat high‐fat diets once born, and therefore, it may be suggested that their mothers may offer some kind of protection for their foetuses, as high‐fat diets during pregnancy in other mammalian and human studies seem to have only negative consequences for metabolic disease risk (Khan *et al*., 2005; Srinivasan *et al*., 2006; Elahi *et al*., 2009). An important recent study in mice has also shown that high‐fat diets during both gestation and lactation result in insulin resistance and obesity in the offspring, with increased inflammation originating in the adipose tissue (Kruse *et al*., 2015). An observation confirmed a background of genetic deletion of gastric inhibitory polypeptide receptor (GIPR) that has previously been shown to prevent high‐fat diet‐induced obesity (Kruse *et al*., 2015). However, if prenatal high‐fat diets are followed by normal diets consumed after pregnancy, it seems that offspring may not develop obesity but type II diabetes *via* increased cytokine activation, inflammation and mitochondrial dysfunction (Latouche *et al*., 2014). These alarming effects of early‐life nutritional stress have culminated in the recent UK guidelines in the importance of maternal weight and diet in influencing the risk of obesity and type II diabetes in humans (NICE 2010).

### Programming in human muscle memory: Evidence from epidemiological studies and relevance to sarcopenia

Together with metabolic disorders, in human aging cohort studies, low birth weight and malnutrition have been associated with the decline in the skeletal muscle mass, low strength and slower gait speed in men and women into old age (Sayer *et al*., 2004b, 2008; Sayer & Cooper, 2005; Patel *et al*., 2010, 2011, 2012, 2014). This leads to the earlier onset of the geriatric disorder known as sarcopenia and is therefore perhaps suggestive that sarcopenia may have origins in foetal and early life. Indeed, the reductions in muscle size and strength have been strongly associated with birth weight independently of height and weight and even when social class, physical activity, smoking and alcohol are adjusted for (Sayer *et al*., 2004b). Furthermore, birth weight has shown a positive association with adult body mass index and fat‐free mass, but not with measures of adult fat mass (Sayer *et al*., 2004a), again indicating that lean body mass reductions in old age, a hall mark of sarcopenia, can be somewhat determined in early life. These impairments in muscle function are associated with earlier onset of disability, morbidity and mortality (Laukkanen *et al*., 1995; Rantanen *et al*., 1999, 2002; Rantanen, 2003; Gale *et al*., 2007). The mechanisms of these early‐life environmental differences and their impact on muscle mass and strength in these cohort studies have begun to be investigated using a cross‐sectional design investigating those with lower vs. higher grip strength or lean body mass. Lower transcript expression of vitamin D receptor and interferon gamma in skeletal muscle tissue was associated with higher lean mass and lower gene expression of interleukin 6 (IL‐6), tumour necrosis factor‐alpha (TNF‐α) and interleukin‐1 receptor (IL1R1) in community‐dwelling older men (Patel *et al*., 2014). Furthermore, lower myostatin was also associated with higher grip strength in community‐dwelling older men, whereas lower IL‐6 in the serum was associated with lower strength (Patel *et al*., 2014), suggesting overall that the inflammation and catabolic/protein degradative regulators are found to be increased in aged cohorts who have reduced strength and lean body mass, observed previously to be correlated with low birth weight. Although epigenetics is considered an important modulator of these foetal origins of metabolism, and studies in other animal models and tissues are beginning to take place, for example reviewed in Saffery (2014), there are limited epigenetic studies into the early‐life origins of sarcopenia. This may be partly due to the expensive nature of the genome‐wide type analysis and access to these human cohorts as well as complex, expensive‐equivalent aging studies in animal models. There is, however, an important study to date that characterizes the methylome and transcriptome of aged, but disease‐free, skeletal muscle vs. young adults (Zykovich *et al*., 2014). These types of studies need to be conducted in the skeletal muscle of maternal nutrient‐restricted offspring into old age and human cohort studies that have early‐life malnourishment or low birth weights in order to distinguish important epigenetic mechanisms of the foetal origins of health and disease. It is also important that future studies assess the epigenetic response of these animals or human cohorts to a later‐life nutrient, muscle growth or loss stimuli in order to address the mechanisms of muscle memory.

## Muscle cellular memory: Evidence from animal tissue models and muscle‐derived stem cells from humans under different environmental niches

### Memory from positive anabolic/growth encounters

In 2010, an important study in mice suggested that the increased muscle nuclei in fibres (myonuclei) acquired *via* a mechanical overload stimulus are retained even when a subsequent period of denervation‐induced muscle loss was encountered vs. controls (Bruusgaard *et al*., 2010). These studies were expanded by the same group in 2013, whereby testosterone was administered to mice for 3 months, enabling robust muscle hypertrophy that was accompanied by an increase in myonuclei (Bruusgaard *et al*., 2010; Egner *et al*., 2013; Gundersen, 2016). These myonuclei were subsequently retained during muscle size losses following a period of testosterone withdrawal. Most importantly, when mechanical overload was presented to the animals that had received an earlier encounter of testosterone vs. controls, they exhibited a 31% increase in muscle cross‐sectional area vs. control mice that showed no growth in the same period of time (Bruusgaard *et al*., 2010; Egner *et al*., 2013). This study therefore alludes to a mechanism whereby muscle has the capacity to adapt to a muscle growth stimulus (overload) with a positive adaptation if hypertrophic stimuli (testosterone) have been encountered previously, in line with our definition of muscle memory in this review. More evidence for memory of positive environments comes from studies that derived muscle ‘stem’ or precursor cells from humans. These studies are important as skeletal muscle tissue is terminally differentiated or postmitotic; therefore, skeletal muscle has its own resident stem cell population known as satellite cells that have mitotic potential and therefore contribute to muscle regeneration, repair and turnover *via* the process of proliferation, migration and fusion into the existing fibres (reviewed in Sharples & Stewart, 2011). Populations of the mitotic cells also self‐renew to allow the replenishment of the stem cell pool for future regenerative bouts (Troy *et al*., 2012). Importantly, a recent study derived these muscle stem cells from active and inactive (sedentary) individuals. The cells derived from active humans displayed an improved ability to uptake glucose and were somewhat protected from palmitate‐induced insulin resistance vs. cells isolated from sedentary humans (Green *et al*., 2013; Valencia & Spangenburg, 2013). These types of cellular studies are also important as they suggest that muscle cells retain molecular information even after isolation outside of their environmental niches. Also, as these cells have mitotic and self‐renewal capabilities, they may be fundamental in passing epigenetic modifications onto daughter populations of cells and therefore involved in regulating skeletal muscle memory, something that is discussed below with respect to epigenetics regulating muscle stem cell self‐renewal into old age.

### Memory from negative catabolic encounters from disease, inflammation and nutrient stress

In the same vein as the studies above regarding the memory of positive environments, earlier work from our group has shown that human muscle stem cells derived from the skeletal muscle bioptic samples of patients with cancer, display memory of the environment from which they were derived. These cells exhibit inappropriate proliferation vs. aged matched cells derived from the muscle biopsies of healthy control patients (Foulstone *et al*., 2003). While at an endocrine level, the IGF system between the groups was not different, at a cellular level, inappropriate IGFBP‐3 expression by the muscle cells derived from the patients with cancer was associated with dysregulated cell retrieval and behaviour (Foulstone *et al*., 2003). These were the first reported studies to identify dysregulated local expression of the IGF system in human skeletal muscle stem cells and associated memory of the derived muscle milieu. Since these earlier studies it has been demonstrated that myotubes grown from muscle‐derived cells isolated from obese humans have greater intramyocellular lipid contents due to the membrane relocation of fatty acid translocase (FAT/CD36) (Aguer *et al*., 2010). More recently, this concept has been confirmed and discussed by four further papers (three original works and one review), again collectively suggesting that muscle‐derived cells retain a ‘memory’ of negative encounters once isolated from different environmental niches, where (i) muscle‐derived cells from obese patients do not respond to lipid oversupply with the same gene expression signatures vs. control (Maples & Brault, 2015); (ii) muscle‐derived cells from intrauterine growth‐restricted sheep foetuses exhibit deficiencies in proliferation vs. controls (Yates *et al*., 2014); (iiii) skeletal muscle stem cells isolated from prenatal 50% global nutrient‐restricted mice offspring or early postnatal high‐fat diet (60% of total calories from fat) offspring have a reduced number of muscle precursor cells and a 32% decrease in the capacity of these cells to regenerate after injury (Woo *et al*., 2011). Interestingly, in the same study it was reported that a high‐fat diet also evoked reductions in the number of muscle stem cells retrieved from skeletal muscle tissue and was associated with larger reductions (44%) in regeneration (chow 16% vs. high‐fat diet 9%), suggesting that the environment in which the muscle cells were derived could be remembered and subsequently affect the muscle repair and regeneration in later life (Woo *et al*., 2011); (iv) finally, the vast majority of aged muscle stem cells isolated from aged animals and humans or those replicatively aged in culture have an impaired or delayed differentiation/regenerative capacity (Allen *et al*., 1982; Charge *et al*., 2002; Lorenzon *et al*., 2004; Lees *et al*., 2006; Carlson & Conboy, 2007; Lancioni *et al*., 2007; Bigot *et al*., 2008; Hidestrand *et al*., 2008; Pietrangelo *et al*., 2009; Beccafico *et al*., 2010; Sharples *et al*., 2010, 2011, 2012; Kandalla *et al*., 2011; Merritt *et al*., 2013; Zwetsloot *et al*., 2013) or display delayed differentiation (Corbu *et al*., 2010). Very few studies showed no impact of age on differentiation of the isolated muscle stem cells (Shefer *et al*., 2006; Alsharidah *et al*., 2013); where even these studies reported aged muscle to have fewer stem cells to begin with (Shefer *et al*., 2006). Furthermore, the ability of these cells to self‐renew, depleting the stem cell pool (Bigot *et al*., 2015), appears to be impaired with age, a process that may be epigenetically regulated (discussed below). Importantly, all of these studies highlight that the epigenetic regulation of muscle ‘stem’ cells with mitotic potential could be important in the concept of muscle memory with respect to metabolic function, muscle repair and regeneration in skeletal muscle tissue.

## Epigenetics underpins programming and gives muscle an ‘Epi‐memory’

Epigenetics translated means ‘above genetics’ and is defined as changes in gene activity and expression as a result of transient or stable structural modifications (primarily to DNA or histones, but also post‐transcriptional modification of RNA) without altering the genetic code. The modifications are caused directly to DNA *via* methylation, or at the level of the surrounding core histones (H)2A, H2B, H3 and H4. Histones have long N‐terminal tails, meaning that they are readily prone to methylation (me), acetylation (ac), phosphorylation (p), ubiquitination (ub), SUMOylation, ADP‐ribosylation and finally citrullination (reviewed in Bannister & Kouzarides, 2011). DNA methylation and histone modification result in alterations in gene transcription. DNA methylation primarily occurs on CpG dinucleotide pairs at the 5′ site of cytosine bases creating 5‐methylcytosine (5mC). This usually leads to gene suppression when occurring within a promoter region of a gene *via* blocking the access of RNA polymerase after the enlistment of methyl CpG binding domain (MBD) proteins (Bogdanovic & Veenstra, 2009). Other DNA methylation that occurs at intragenic sites, however, has an extremely variable impact on gene transcription, only when intragenic methylation leads to repressing the function of long‐range gene enhancers and it has been shown to more consistently suppress gene expression (Weber *et al*., 2007; Schmidl *et al*., 2009), and intragenic methylation can also be involved in modulating alternative splicing of the gene (Shukla *et al*., 2011; Sati *et al*., 2012; Maunakea *et al*., 2013). The process of DNA methylation and demethylation is controlled by DNA methyltransferases (DNMTs) and demethylases, called the ten‐eleven translocation (TET) enzymes. De novo methylation is controlled by methyltransferases DNMT3a and DNMT3b, whereas DNMT1 maintains methylation (Trasler *et al*., 2003). Once methylation is not required, TET1, 2 and 3 convert the 5mC to 5‐hydroxymethyl cytosine (5hmC) for the subsequent removal *via* base excision repair (Tahiliani *et al*., 2009; Ito *et al*., 2010). Histone modifications, however, are more complex than DNA methylation in their action to activate or suppress gene transcription. Generally, histone methylation of lysine 4 of histone H3 (H3K4me3) is associated with an increased gene expression as is acetylation of numerous lysine residues of histones H3 and H4 (acetyl H3 and acetyl H4), whereas trimethylation of lysines 9 and 27 of histone H3 (H3K9me 2/3 and H3K27me3) and lysine 20 of histone H4 (H4K20me3) is involved in gene suppression (reviewed in Schuettengruber *et al*., 2011). Acetylation of histones is controlled by the histone acetyltransferases (HATs) such as K‐Lysine acetyltransferase 2A (KAT2A or GCN5), P300/CBP‐associated factor (PCAF), CREB‐binding protein (CBP), p300, K‐Lysine acetyltransferase 5 (Tip60) and male absent on the first (MOF), that add acetyl groups to/from target histones. In contrast, deacetylases (HDACs) remove acetyl groups, including the class I (HDAC 1‐3 and 8), class II (HDAC 4–7, 9–10), class III (Sirt1‐7) and class IV HDACs (HDAC11). As described above, acetylation of histones leads to active transcription, and HATs therefore have been associated with active gene expression and the HDACs gene suppression (reviewed in Wang *et al*., 2009). For the purposes of this review, the above summarized mechanisms are currently those that are studied with respect to skeletal muscle memory. For a more detailed role of histone modifications in modulating gene expression, this is reviewed extensively in Bannister & Kouzarides (2011).

Despite epigenetic modifications underpinning gene expression operating following changes in environmental stimuli (reviewed in Bhutani *et al*., 2011), there are few studies to investigate the potential epigenetic modulation of skeletal muscle cell memory. Currently, there are more extensive data that exist regarding the epigenetic control of muscle stem cells and the process of myogenesis and muscle stem cell function (reviewed in Dilworth & Blais, 2011; Segales *et al*., 2015; Laker & Ryall, 2016), including work by ourselves on the class III HDAC sirtuin 1 (Saini *et al*., 2012). However, a very recent study has investigated the underlying epigenetics in muscle‐derived cells of obese patients where lipid oversupply in these cells induced increased DNA methylation of PPARδ with the subsequent greater suppression of PPARδ gene expression vs. nonobese controls (Maples & Brault, 2015). Despite the subjects in the control group not being aged matched with the obese group, where the obese group were on average almost 7 years older, this study implies that epigenetic modification of lipid metabolism genes after encountering a negative metabolic environment (obesity) was retained once isolated in culture, and that epigenetic regulation of lipid metabolism genes were important mediators of metabolic function when a similar later‐life environment (lipid oversupply) was encountered. At the tissue level, another study to investigate the potential epigenetic mechanisms underpinning metabolic programming as a consequence of maternal protein restriction vs. controls (8% vs. 20%) suggested that the reduction in PGC‐1α gene expression in the offspring's skeletal muscle was underpinned by increases in its own promoter methylation (Zeng *et al*., 2013). Furthermore, early (postnatal) high‐carbohydrate diets reduced Glut4 transcription in the skeletal muscle of the adult offspring (100 days postgestation) *via* epigenetic modifications including an increased methylation of the Glut4 promoter and acetylation of H2 variant (H2A.Z) and H4, importantly highlighting a novel epigenetic mechanism contributing to early‐life nutrient stress that is associated with later‐life adult insulin resistance and obesity (Raychaudhuri *et al*., 2014). In humans, the relationship between nutrient stress and the potential epigenetic modulation of diabetes and obesity was also investigated where only 5 days of a high‐fat feeding (50% extra calories distributed as 60% fat vs. controls with no extra calorie consumption and diet made up of 35% fat) reportedly altered DNA methylation of over 6508 genes of 14475 genes studied (Jacobsen *et al*., 2012). Interestingly, there was a slow reversibility of DNA methylation when the high‐fat diet was switched back to the control diet for 6–8 weeks, where out of the top 10% of altered genes post the high‐fat diet, only 66% of these genes had a methylation status in the opposite direction, with only 5% of these significant (Jacobsen *et al*., 2012). Importantly this study suggests that methylation could be retained and the implication that CpG methylation could perhaps be preserved or built up over time after multiple high‐fat diet periods. De novo vs. maintained methylation, as discussed above, is controlled by DNMTs that also increased post the high‐fat diet in the same study, where both de novo regulating DNMT3a and maintaining DNMT1 mRNA increased (Jacobsen *et al*., 2012). The authors did not study the activity of these enzymes that may also underpin the maintenance of methylation some 6–8 weeks later.

Importantly, our group has shown that skeletal muscle cells have an increased susceptibility to the impaired differentiation after encountering TNF‐α inflammatory stress in later proliferative life if the cells have experienced an earlier, acute TNF‐α stress (Sharples *et al*., 2015a), suggestive of a morphological memory of catabolic stress in myoblasts. Importantly, in this study, the muscle cells that experienced even an acute cytokine stress in early life retained elevated myoD methylation even after 30 population doublings (Sharples *et al*., 2015a). This study therefore suggests an important epigenetic mechanism *via* the retention of DNA methylation of important myogenic regulatory genes throughout proliferative lifespan in the muscle cells following early‐life inflammatory stress. The de novo and maintenance of this methylation *via* modulating DNMTs and TETs now requires further investigation in this model. It has previously been shown in developing xenopus embyo's that memory of myoDs active gene state can persist through 24 cell divisions in the absence of transcription (Ng & Gurdon 2008). Interestingly, rather than methylation of the myoD promoter, this epigenetic memory was regulated by the association of histone H3.3 with the myoD promoter (Ng & Gurdon 2008), where histone H3.3 requires future investigation in adult muscle cell models of memory such as those described by Sharples *et al*., (2015a) above. As chronic TNF‐α is increased in the circulation (Greiwe *et al*., 2001; Bruunsgaard & Pedersen, 2003; Bruunsgaard *et al*., 2003a,b) and in that produced by skeletal muscle tissue in aged individuals (Greiwe *et al*., 2001; Leger *et al*., 2008) as well as being associated with sarcopenia and muscle loss (reviewed in (Saini *et al*., 2006; Sharples *et al*., 2015b), these studies suggest a potential important mechanism for muscle memorys involment in muscle loss with age. Furthermore, recent pioneering research deriving aged human muscle stem cells showed impairments in the ability of these cells to self‐renew (Bigot *et al*., 2015), as described above, a process that is required to replenish the stem cell pool across the lifespan. In this study was reported that elderly muscle stem cells once isolated in culture demonstrated significantly lower proportion of reserve (PAX7‐expressing) cells and an impaired ability for these cells to be incorporated as satellite cells when engrafted back into immunodeficient mice vs. young human muscle stem cells (Bigot *et al*., 2015). Importantly, these cells also had higher global DNA methylation (assessed *via* whole DNA methylome array). Importantly, by using a demethylation agent, 5‐aza‐20‐deoxycytidine (5AZA) in culture, aged muscle stem cells improved their self‐renewal capacity. Of particular interest, the authors reported that increased DNA methylation of sprouty1 (or SPYR1 a known regulator of self‐renewal/quiescence) corresponded with the reduced SPRY1 transcription (Bigot *et al*., 2015). By knocking down SPYR1 to 5% using small interfering RNA (siRNA), even in the presence of global demethylation *via* 5AZA treatment, the capacity to self‐renew in these cells was completely abolished, confirming the important role of epigenetically regulated SPRY1 in the loss of the muscle stem cell pool with age. This manuscript now opens up the possibility of epigenetic characteristics being ‘reset’ in aged muscle to restore normal function where self‐renewal is fundamental to the capacity of muscle to respond to repeated regenerative bouts across the lifespan, demonstrating a clear clinical translation. Earlier work also suggested that increased senescence in isolated muscle stem cells from aged mice, characterized by an increased in p16INK4, was controlled epigenetically by the polycomb protein 1/H2A ubiquitination (PRC1/H2Aub) and the subsequent loss in repressive function of P16INK4a gene expression (Sousa‐Victor *et al*., 2014), importantly, also suggesting that, together with DNA methylation, histone modifications can also be retained by muscle cells once isolated from their niches that may subsequently affect their function into old age. Overall, these studies strongly suggest that epigenetic modifications are retained throughout the lifespan and can affect skeletal muscle function in later life. Importantly, these types of epigenetic modifications could be targeted therapeutically in future to ‘reset’ skeletal muscle loss associated with aging or perhaps even diseases such as cachexia.

### Epigenetics of exercise adaptation and exercise interventions as a suitable model to investigate skeletal muscle memory

Muscle memory originally entered scientific literature synonymous to motor learning, where the learning of a motor task involves consolidating a specific movement pattern through repetition. Within exercise or strength and conditioning settings, muscle ‘memory’ is largely an anecdotal phrase used to describe the ability of adult human skeletal muscle to respond more advantageously to stimuli that have already been encountered in the past, where applied practitioners often describe the ability of individual muscle to respond more quickly, for example after injury or the off‐season, to an environmental stimulus, such as a period of resistance or aerobic training that has already been experienced in the past. There is currently evidence that muscle responds morphologically and functionally differently following retraining (i.e. following an earlier period of exercise and cessation/detraining of exercise), especially postresistance exercise (Staron *et al*., 1991; Taaffe & Marcus, 1997; Henwood & Taaffe, 2008; Taaffe *et al*., 2009), that myonuclei are retained after prior anabolic hormonal/exercise encounters (Bruusgaard *et al*., 2010; Egner *et al*., 2013; Gundersen, 2016) and finally that epigenetic modification occurs in the skeletal muscle following exercise (reviewed in Ntanasis‐Stathopoulos *et al*., 2013; Goto *et al*., 2015). However, the epigenetic mechanisms for exercise‐induced muscle memory still require further investigation.

Most notably, with respect to exercise‐induced epigenetic adaptations, 60 min of acute high‐intensity cycling exercise (75% VO_2_ max/maximal aerobic capacity) induced histone acetylation of H3 in skeletal muscle, a process that was found to be controlled by the removal of the HDACs from the nucleus (McGee *et al*., 2009). Furthermore, following initial studies suggesting that PGC‐1α hypermethylation resulted in associated reductions in mitochondrial content in patients with type 2 diabetes (Barres *et al*., 2009), it has subsequently been observed that there was reduced DNA methylation of PGC‐1α, mitochondrial transcription factor A (TFAM) and pyruvate dehydrogenase lipoamide kinase isozyme 4 (PDk4) (all associated with mitochondrial biogenesis) immediately post acute aerobic exercise at 80% of maximal aerobic capacity (until 1674 KJ was expended) and a reduction in PPARδ methylation 3 h post exercise, corresponding with reductions in their own gene expression (3 h post exercise for PGC‐1α, PDK4 and PPAR‐δ, immediately post for TFAM) (Barres *et al*., 2012). Lower exercise intensities (40% vs. 80% VO_2_ max until 1674 KJ was expended) were, however, found to evoke no methylation changes in the above‐mentioned genes (Barres *et al*., 2012). More, recently 120 min of steady‐state exercise (60% Vo_2_ peak) reportedly increased methylation in the promoter of fatty acid‐binding protein 3 (FABP3) after 4 h of recovery resulting in the impaired mRNA transcription (Lane *et al*., 2015). Furthermore, suppression of DNA methylation was also associated with increased basal mRNA levels of the PGC‐1α promoter A after exercise but not the PGC‐1α promoter B where this was controlled by the methylation of lysine 4 on histone 3 (H3K4me3) (Lochmann *et al*., 2015). With respect to chronic exercise, 6 months of supervised aerobic exercise 3 × 1 h per week (varied intensity) changed the methylation of several genes associated with metabolism (Nitert *et al*., 2012). These included increases in DNA methylation of runt‐related transcription factor 1 *(RUNX1)* and myocyte‐specific enhancer factor 2A *(MEF2A)*, transcription factors involved in exercise‐induced changes in Glut4 expression influencing glucose uptake in skeletal muscle (Smith *et al*., 2007, 2008). Furthermore, in the same study, methylation of NADH dehydrogenase ubiquinone 1 subunit C2 (*NDUFC2)* increased*,* encoding an enzyme that is part of the respiratory chain (Olsson *et al*., 2011), as well as observed alterations in the methylation of adiponectin receptors *1* and 2 and bradykinin receptor *(BDKRB2)*, which are both involved in regulating metabolism in the skeletal muscle (Taguchi *et al*., 2000; Yamauchi *et al*., 2003). Together with epigenetic alterations at the tissue level, epigenetic modifications are observed in satellite cells after exercise‐induced activation (Fujimaki *et al*., 2014). Increased Wnt/beta‐catenin signalling post exercise caused histone modifications promoting gene activation such as H3K4me2 and H3Ac together with corresponding decreases in gene suppressing H3K9me2 on MyF5 and myoD gene promoters that subsequently led to observed exit from the quiescent states in satellite cells and increased number of dividing satellite cells post exercise (Fujimaki *et al*., 2014). Most notably, exercise in obese mothers (mice) rescued hypermethylation of PGC‐1α and corresponding reductions in mRNA levels of PGC‐1α, Glut4, Cox4 and CytoC together with the loss in metabolic function in later life of the offspring that was a consequence of the obese *in utero* environment in the nonexercise group (Laker *et al*., 2014). Collectively, the above studies fascinatingly suggest that both acute and chronic exercise stimuli can cause epigenetic modifications in skeletal muscle, with the later studies suggesting that an exercise stimulus in the parents can be ‘remembered’ by the offspring. However, it remains to be seen whether these exercise‐induced methylation changes can be retained, or over what period they are lost before a further acute or chronic exercise encounter is required to have the same or accumulative effect. There are currently little or no studies into epigenetic modulation post acute anabolic stimuli such as that of acute resistance exercise or those induced by muscle hypertrophy *via* chronic resistance exercise. To the authors’ knowledge, there are also no studies investigating the epigenetic mechanisms underlying muscle adaptation to prior encounters with muscle growth or loss associated with resistance exercise or metabolic adaptation associated with aerobic exercise. Importantly, studies are required to understand skeletal muscle memory of muscle loss encounters associated with disuse or inactivity, or following nutrient feeding, for example via amino acid administration, or nutrient stress, for example CHO restriction or calorie restriction. Finally, it is unknown whether the skeletal muscle can retain any increase or suppression of methylation if a similar exercise stimulus is encountered in later life, or more stably retain these changes following chronic exercise. As exercise stimuli are routinely undertaken in human skeletal muscle adaptation studies, we propose that exercise *via* exposure to ‘earlier life’ training followed by the cessation of training (detraining) back to baseline and then followed by subsequent retraining would be a suitable model to investigate the epigenetic adaptation of skeletal muscle memory at the tissue and cellular level. Finally, it will be fundamental to experimentally determine the importance of epigenetic modifications using the memory evoking models described above in both terminally differentiated muscle fibres/tissue and satellite cells that do have mitotic potential. As muscle tissue is postmitotic, it could be assumed that epigenetic modifications would be more transient and perhaps important for more acute adaptation. On the other hand, satellite cells can proliferate, self‐renew and potentially pass on epigenetic modifications to daughter populations (Sharples *et al*., 2015a); therefore once incorporated into tissue as myonuclei, this would perhaps implicate a role for satellite cells in memory of the muscle tissue later in life. These notions require important future attention to determine the epigenetic adaptation of skeletal muscle memory and the importance of these modifications both at the cellular and tissue level (depicted in Fig. 1).

## Conclusion

Skeletal muscle memory is defined as *‘*The capacity of skeletal muscle to respond differently to environmental stimuli in an adaptive or maladap‐tive manner if the stimuli have been previously encountered.’ Based on the evidence from metabolic programming studies in animal models, epidemiological evidence in humans as well as a suggested retention of epigenetic information in skeletal muscle cells isolated from different environmental niches or over daughter populations, we suggest that; memory in skeletal muscle is underpinned by epigenetic modifications (epi‐memory). The future study of epigenetic modifications during periods of muscle growth, loss, and regrowth, as well as during metabolic disease and aging across the lifespan, has the potential to pave the way for future understanding into the mechanisms of skeletal muscle adaptation and plasticity and potentially provide novel therapeutic targets for ameliorating muscle loss conditions.

## Funding info

No funding information provided.

## Conflict of interest

None declared.
